# Supplementary material for: The challenge of phasing-out fossil fuel finance in the banking sector
Source: Nat Commun. 2024 Sep 10;15:7881. doi: 10.1038/s41467-024-51662-6 (PMC11387497; doi:10.1038/s41467-024-51662-6)
Supplement: Supplementary file 1 — Supplementary Information [file 41467_2024_51662_MOESM1_ESM.pdf]

The challenge of phasing out fossil fuel finance in the banking sector

Supplementary Information

J. Rickman[1], M. Falkenberg[2], S. Kothari[1], F. Larosa[3], M. Grubb[1], N. Ameli[1]

[1]Institute for Sustainable Resources, University College London, UK

[2] Department of Mathematics, City University of London, London, UK

[3] KTH Royal Institute of Technology, Sweden

Supplementary materials containing supplementary figures, tables and methods.

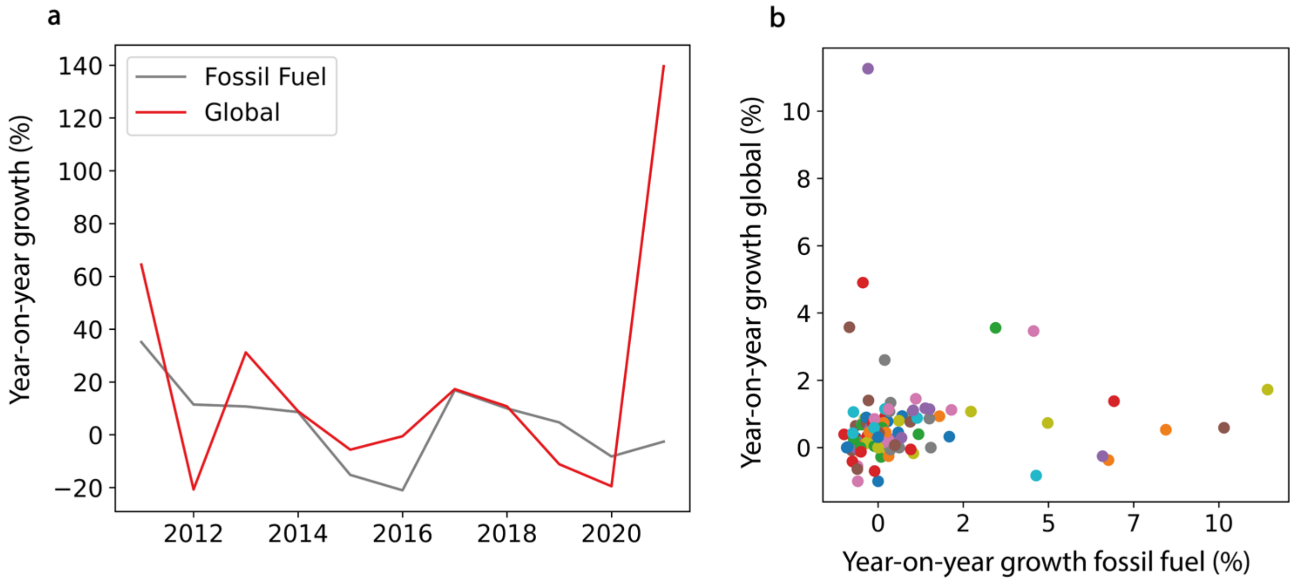

Figure S1. **Comparing activity in the fossil fuel and global syndicated debt market.** (a) Line plot showing year-on-year growth rate in the global (red line) and fossil fuel (grey line) syndicated debt market between 2011 and 2021. (b) Scatter plot showing year-on-year growth in the fossil fuel syndicated debt market against year-on-year growth in the global syndicated debt market for the top 100 banks in the fossil fuel debt market, ranked by cumulative fossil fuel investment.

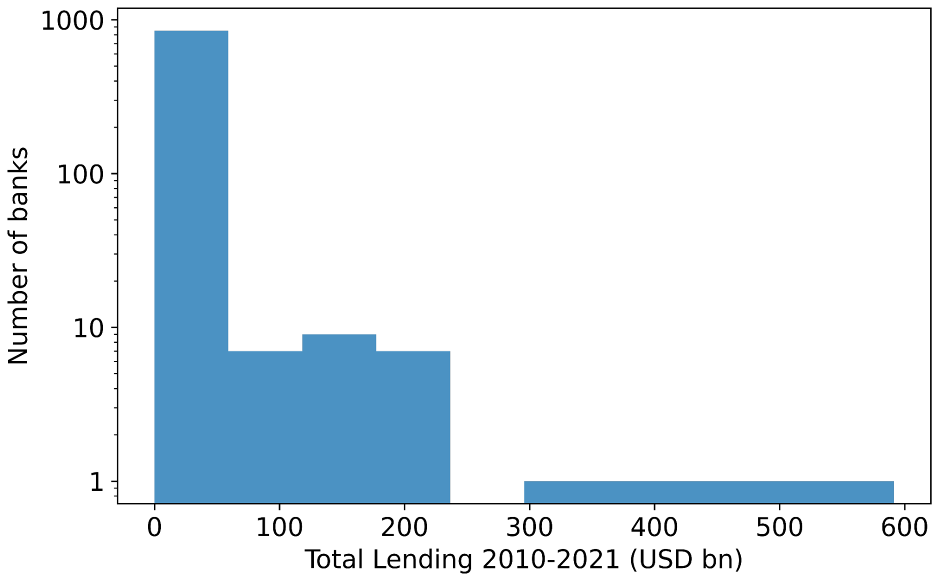

Figure S2. **Lending activity across the banking sector.** Histogram shows the total lending of 709 banks between 2010 and 2021.

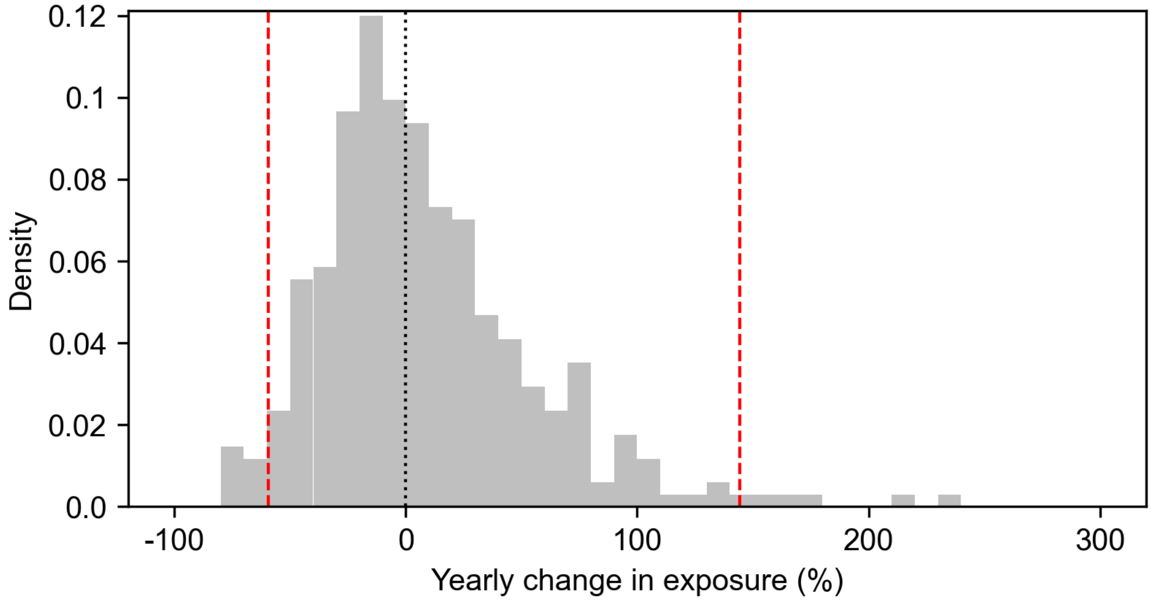

Figure S3. **Year on year change in fossil fuel exposure for the top 30 banks.** Data is aggregated across the period 2010 to 2021. Dashed black line corresponds to 0% change in exposure. Dashed red lines indicate 95% confidence interval.

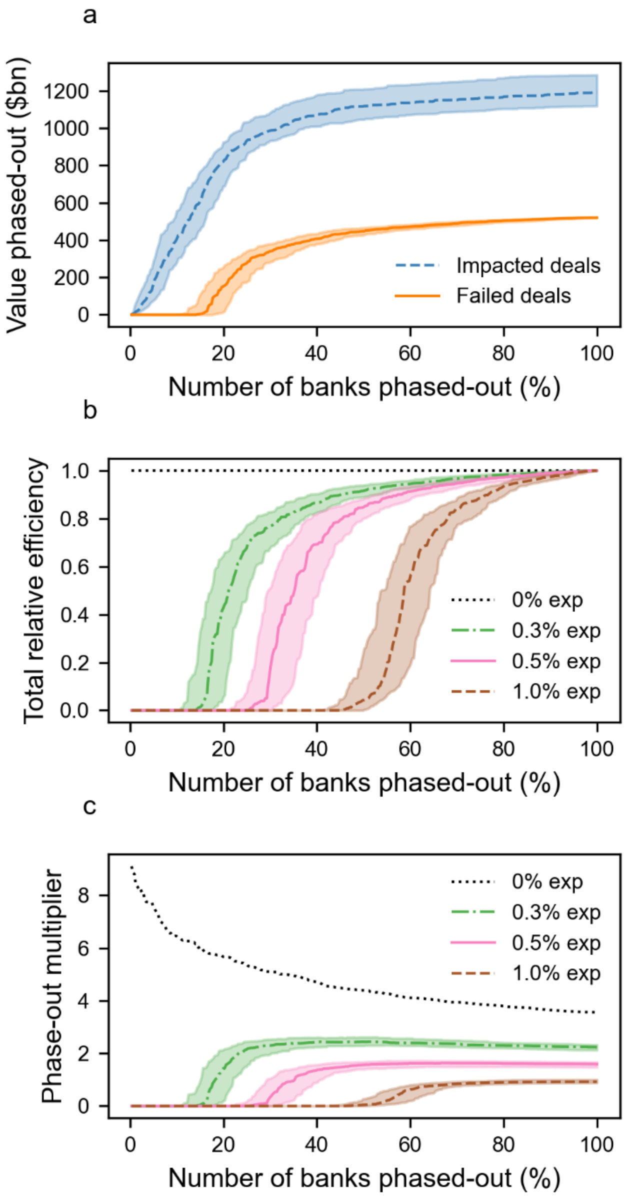

Figure S4. The phase-out model using absolute exposure as a percentage of total assets as the finance limit. (a) Banks exit the fossil fuel sector in a random order (321 banks total). The cumulative value of impacted deals using an exposure limit of 0.3% (fossil fuel assets as a percentage of all assets; blue dashed); this includes both the deals which do, and do not, succeed in acquiring substituted finance. The cumulative value of the deals which fail (orange solid). Note that a single deal can count towards the cumulative impacted and failed deals multiple times, once for each time a syndicate partner pulls finance from the deal; conversely, a single deal can only fail once. (b) The total relative phase-out efficiency (see methods for definition) for various exposure limits in the phase out model. If few banks exit the sector, efficiency is low since deals successfully substitute finance. Beyond a tipping point, efficiency grows rapidly. (c) The total phase-out multiplier (see methods for definition) for various difference exposure limits. A multiplier larger than 1 indicates that the direct phase-out of finance by one bank has forced the indirect phase-out of finance from syndicate partners. All curves correspond to the median value across 100 random simulations. Shaded regions correspond to inter-quartile range across the 100 simulations. These results are qualitatively comparable to the results shown using the finance limit percentage in Figure 3 of the main text.

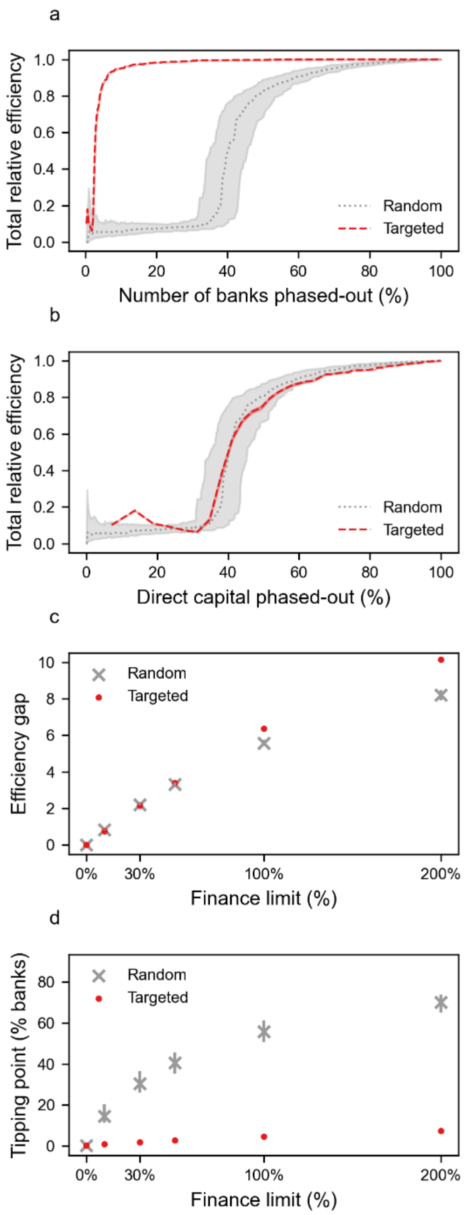

Figure S5. Comparing the targeted and random scenarios using the syndicate substitute rule for finance substitution. (a) Total relative efficiency as a function of the percentage of banks which have phased-out from the fossil fuel sector. Targeted removal of banks (red dashed) reduces the number of banks which must be phased-out to achieve non-zero efficiency, relative to the random case (grey dotted). For illustrative purposes, both scenarios use a capital limit of 100%. (b) The equivalent to panel (a), but as a function of the total capital phased-out directly by banks. (c) The efficiency gap for various different finance limit percentages. In contrast to the any substitute scenario, for a fixed limit the efficiency gap is larger for the targeted phase-out (red points) than the random phase-out (grey crosses). (d) The tipping point required to achieve a total relative efficiency of 0.5. All panels show the median value from 100 simulations for each model. Shaded regions correspond to inter-quartile range across the 100 simulations.

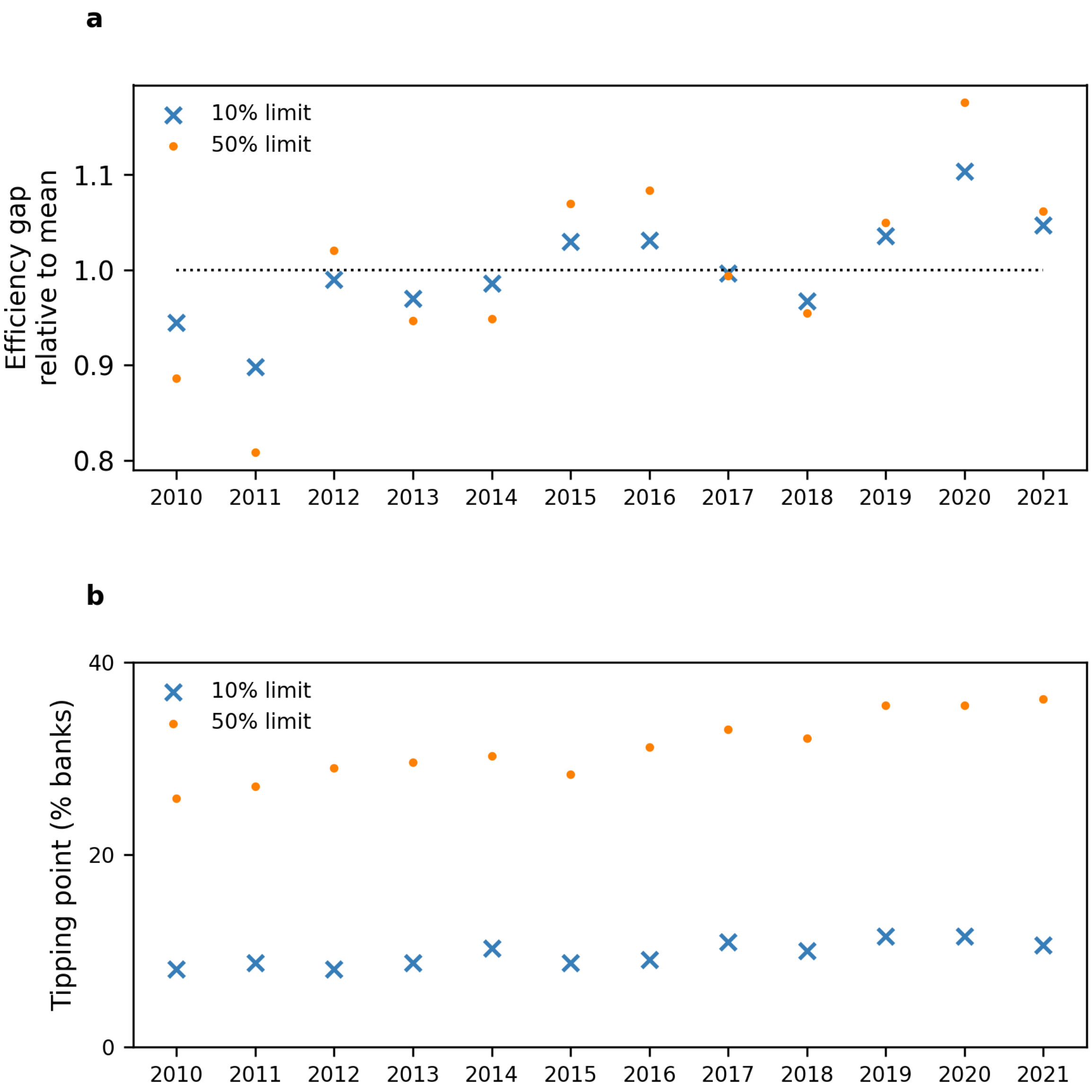

Figure S6. Efficiency gap and tipping point each year. (a) The size of the efficiency gap as a multiple of the average efficiency gap in the period 2010-2021. The efficiency gap is relatively stable for a fixed finance limit over time, fluctuating between +/-20% in the observed time period. Larger finance limits result in larger fluctuations in the efficiency gap. (b) The percentage of banks that must be removed to reach the tipping point for each year in the period 2010-2021 for two different finance limits. Note that the tipping point is larger for the 2021 syndicated network than for any previous year, despite not having the largest efficiency gap. This is a consequence of growing deal syndication between 2010-2021.

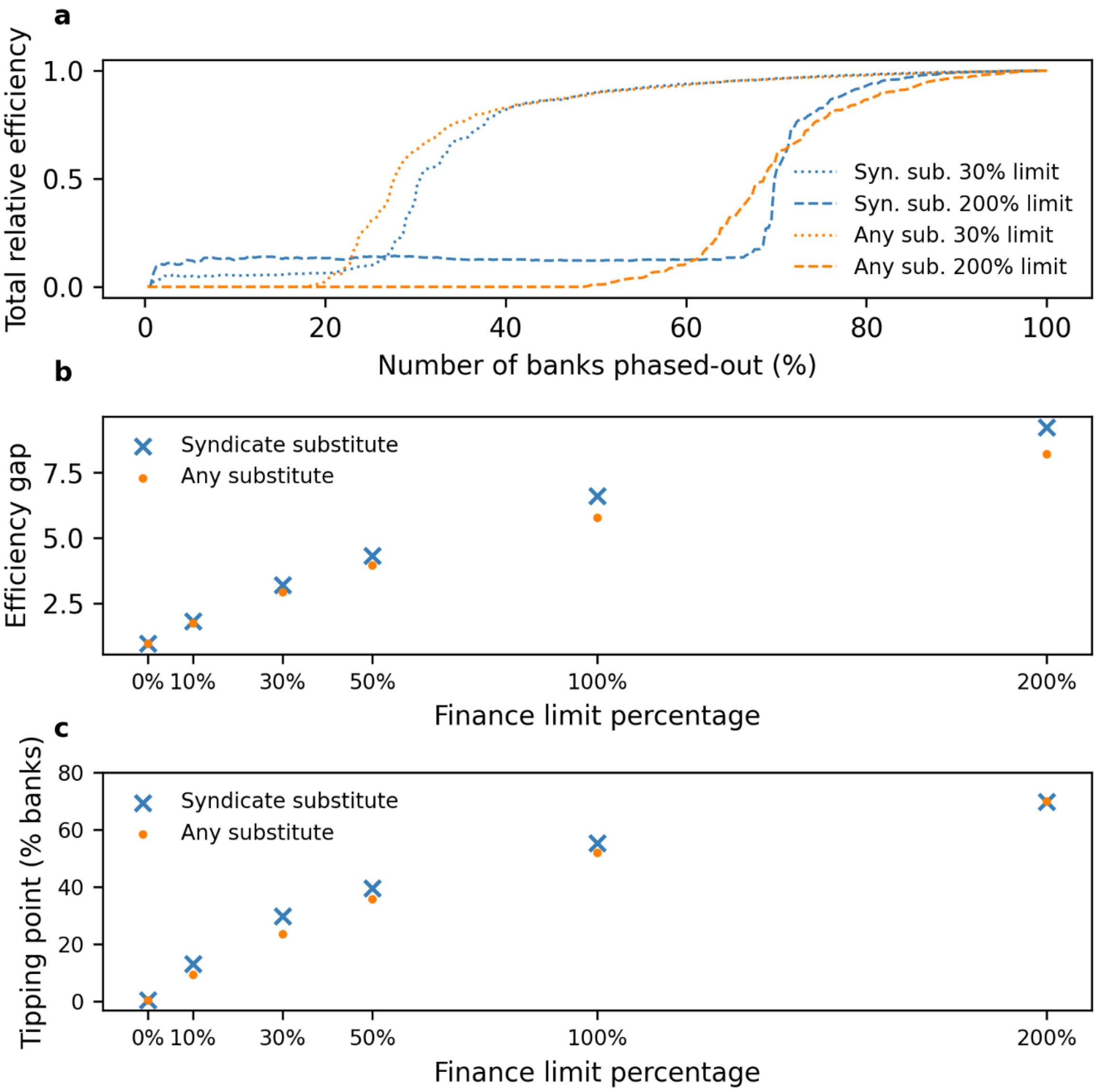

Figure S7. Phase-out model variant comparing the any substitute and syndicate substitute models. (a) Banks exit the fossil fuel sector in a random order (321 banks total). The relative efficiency of the phase-out process for two different finance limit percentages. (b) The efficiency gap for the any substitute and syndicate substitute models with different finance limits. (c) The tipping point the any substitute and syndicate substitute models with different finance limits.

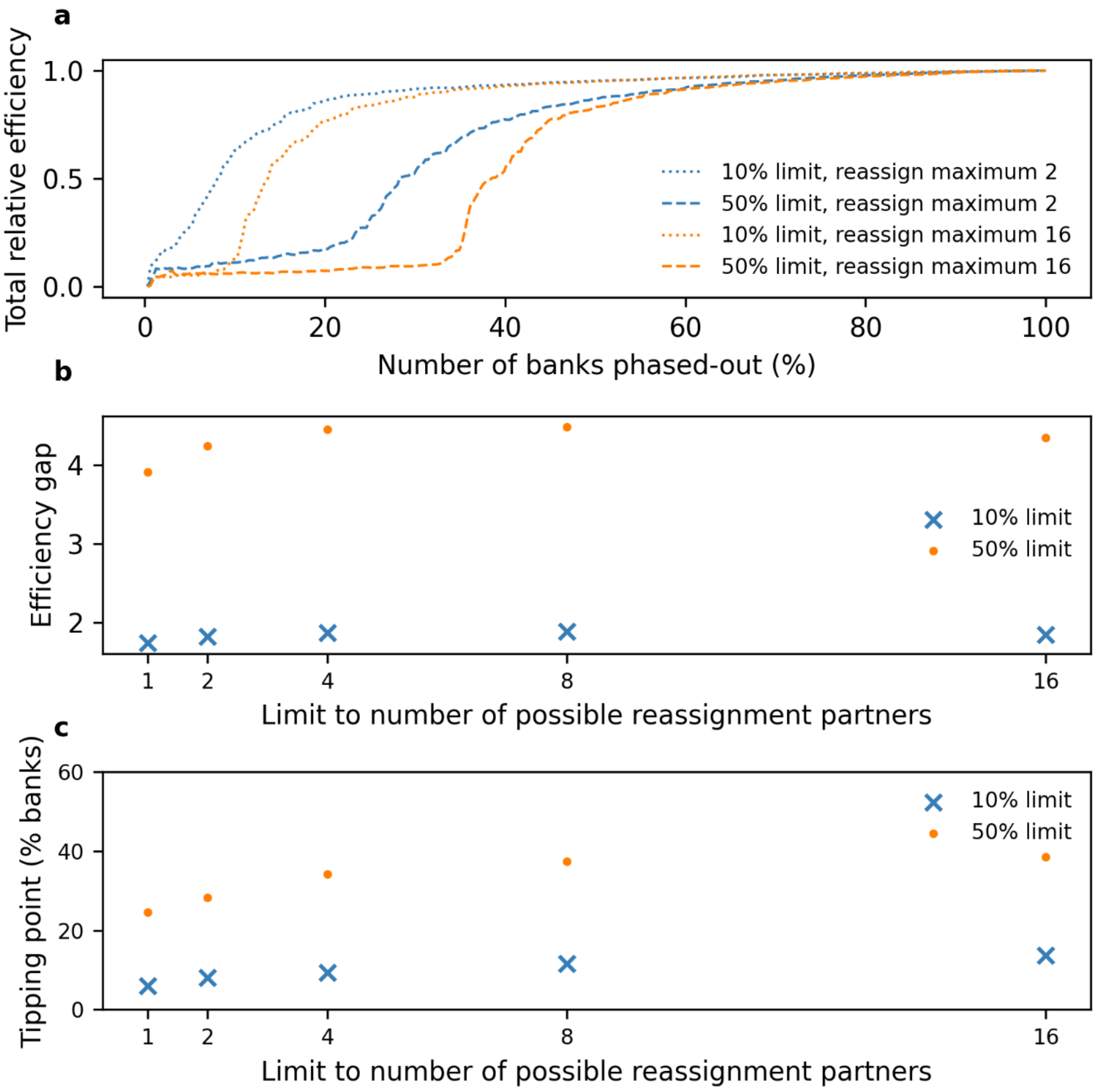

Figure S8. Phase-out model variant with restrictions on the maximum number of banks that can substitute finance into a deal at risk. All simulations carried out using the syndicate substitute model variant. (a) The total relative efficiency with two different finance limit percentages (10% and 50%) and two values of the bank substitute partner limit (2 and 16 for illustrative purposes), which restricts the number of candidate banks from whom substitute finance can be acquired. (b) The efficiency gap as a function of the finance limit and the bank partner reassignment limit. (c) The tipping point as a function of the finance limit and the bank partner reassignment limit.

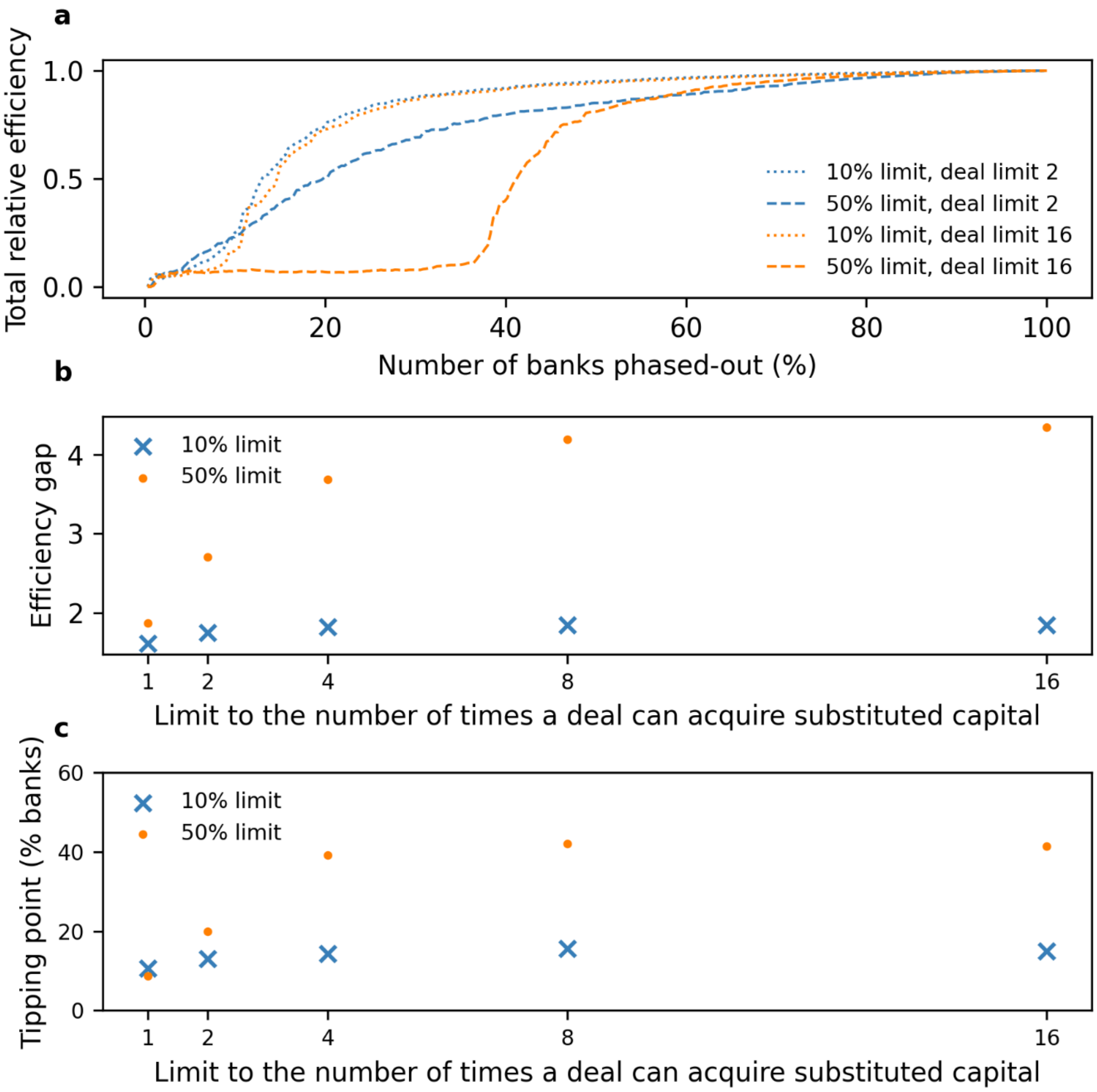

Figure S9. Phase-out model variant restricting the number of times a single deal can acquire substituted finance before automatically failing. All simulations are carried out using the syndicate substitute model variant. (a) The total relative efficiency with two different finance limits (10% and 50%) and two values for the finance substitution limit (2 and 16 for illustrative purposes), which restricts the number of times a single deal can acquire substituted finance after a partner bank phases-out from the fossil fuel sector, before the deal automatically fails. (b) The efficiency gap as a function of the finance limit and the deal substitution limit. (c) The tipping point as a function of the finance limit and the deal substitution limit.

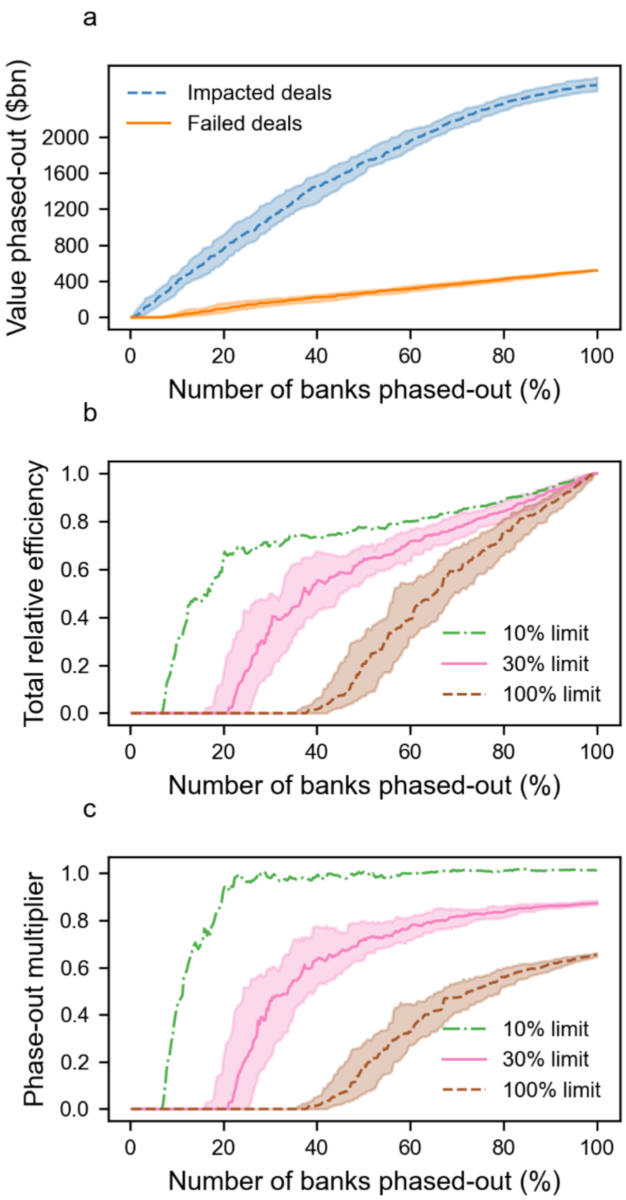

Figure S10. The phase out model using the any substitute rule and with banks phased-out in a random order, but where finance freed up from active banks who were syndicate partners in a failed deal can be reassigned to new deals. (a) Banks exit the fossil fuel sector in a random order (321 banks total). The cumulative value of impacted deals using a finance limit of 10% (blue dashed). The cumulative value of the deals which fail (orange solid). Due to the reassignment of finance from failed deals, the efficiency gap is larger in this scenario than in the scenario where finance cannot be reassigned from failed deals to new deals. (b) The total relative phase-out efficiency (see methods for definition) for various different finance limits in the phase out model. If few banks exit the sector, efficiency is low since deals successfully substitute finance. Beyond a tipping point, efficiency grows rapidly. (c) The total phase-out multiplier (see methods for definition) for various difference finance limits. Due to the reassignment of finance, direct phase-out cannot induce the indirect phase-out of capital in this scenario. All curves correspond to the median value across 100 random simulations. Shaded regions correspond to inter-quartile range across the 100 simulations.

Centrality Measure	Pre-Paris (2010-2016)	Post-Paris (2017-2021)
Degree	0.98	0.99
Eigenvector	0.98	0.98
Closeness	0.34	0.35
Betweenness	0.63	0.61

Table S1. Correlation between banks' centrality and syndication activity. Pearson's correlation coefficient between banks' syndication activity and network centrality measures in a pre-Paris Agreement (2010-2016) and post-Paris Agreement (2017-2021) period. The weighted versions of eigenvector and betweenness centrality are computed.

Supplementary Methods

Data coverage

Comparing the Bloomberg data with a study by the European Commission[1] sourcing data from alternative industry leading providers of financial data, Refinitiv and Thomson Reuters, affirms Bloomberg provides excellent coverage of global bank debt provided to the fossil fuel sector. The European Commission reports 2.12 trillion USD of debt provided between 2016 and 2020 compared to 2.9 trillion USD reported by Bloomberg. The dataset was further augmented by identifying each bank's ownership status (public or private) through Google search and verified through the Bloomberg terminal where necessary. Public banks, defined as banks that are majority owned by one or multiple government entities, provided just 6% of finance over the period analysed.

Phase-out model results for the years 2010-2021

Figure S3 shows the yearly change in fossil fuel exposure for the top thirty banks in our dataset, measured in terms of absolute value of fossil fuel deals underwritten during the period. Data is aggregated across the period 2010 to 2021. The figure shows that the modal annual change in fossil fuel exposure during this period is a small annual decrease. However, there are many cases where banks significantly increase their fossil fuel exposure, in some cases by over 100%. The 95% confidence interval for the annual change in fossil fuel exposure for top banks falls in the range [-60%,+160%].

Figure S4 shows results for the phase-out model computed with data from 2010 to 2021. Banks are phased-out in a random order, and candidate banks for substitution are chosen using the any substitute rule. For each year, we compute the efficiency gap and tipping point using two different values of the finance limit percentage. In Fig. S4a we plot the efficiency gap for both finance limits (10% and 50% for illustrative purposes) relative to the mean efficiency gap across all 12 years. We find that the efficiency gap only varies by small amounts over time for fixed finance limit percentages. In years with greater fossil fuel lending and syndication, the efficiency gap is generally larger than in years where lending is lower. These effects are larger for higher finance limit percentages. However, overall fluctuations in the finance limit are small, with the efficiency gap always within 20% of the mean value across the time period.

In terms of the tipping point, we see that our result that the tipping point is delayed for larger finance limits is robust across all 12 years studied. We note that although the efficiency gap has fluctuated over time, reaching a peak in 2020, the tipping point has steadily grown over time. This is indicative of the growing level of syndication of the fossil fuel lending network in the period 2010-21.

Variants of the phase-out model

We now study four variants of the basic phase-out model. First, we study how changes to the procedure for finding new syndicate partners for substitution affect fossil fuel phase-out (Fig. S5). This expands on the results shown in the main text in Figure 5a. In the 'any substitute' variant, any active bank who is not already part of the current syndicate and has not exceeded their finance limit percentage can be chosen as a candidate for finance substitution. In the 'syndicate substitute variant' finance is replaced by banks ranked according to the number of deals in which they are co-active with the current deal-at-risk's syndicate. Banks already part of the syndicate are not included. We also compare the random and targeted scenarios using the syndicate substitute rule, see Figure S6, expanding on the results from Figure 4 of the main paper.

Second, we consider how limits to the number of banks who can act as candidates for substitution in the syndicate substitute model variant affects capital phase-out (Fig. S7). This expands on the results shown in Figure 5b of the main manuscript.

Third, we test a model variant that accounts for the increased perceived risk of a deal that acquires substitute finance multiple times, by limiting the number of times a deal can substitute finance before automatically failing (Fig. S8). This expands on the results shown in Figure 5c of the main manuscript.

Fourth, we test an alternative to the finance limit percentage as a cap to finance substitution. In this scenario, the cap is set as an absolute limit of exposure as a percentage of total assets (Fig. S9). Note that we only have exposure values for the top thirty banks in the sector. Therefore, we model the exposure values for other banks providing fossil fuel finance by using a gamma distribution which is fitted to the exposure distribution for the top thirty banks. This model variant shows qualitatively equivalent behaviour to the model variant using the finance limit percentage in Figure 3 of the main manuscript.

Finally, we consider the scenario in which finance from a failing deal can be reassigned to new deals by banks which remain active in the sector (Fig. S10). This is in contrast to the base model in which this finance is indirectly phased-out if the deal fails. This model variant is qualitatively equivalent to the scenario in which finance cannot be reassigned once a deal fails, however, the reassignment of capital means that direct phase-out cannot induce indirect phase-out, such that the phase-out multiplier is always smaller than 1.

The results from model variants are discussed in more detail below.

(i) Syndicate substitute vs. any substitute scenario.

In the base model, candidate syndicate partners for substitution are chosen at random from all banks who are not phased-out from the sector and who are not yet part of the syndicate. We refer to this as the ‘any substitute’ case. We now compare this to a model where syndicate partners are chosen preferentially in order of the total number of syndicates in which the existing syndicate banks co-invest with the candidate bank. We refer to this as the ‘syndicate substitute’ case. Results are shown in Fig. S5 using two different values for the finance limit percentage, 30% and 200% for illustrative purposes.

In Fig. S5a, we see two key differences between the any substitute and syndicate substitute cases: (1) In the syndicate substitute case, there is non-zero phase-out efficiency before the tipping point is reached (efficiency > 0.5. However, (2) this increase in early phase-out efficiency appears to delay the onset of the tipping point. Overall, we see in Fig S5b&c that there is little difference in the efficiency gap and tipping point that arises between the two cases for fixed finance limits.

Figure S6 compares the random and targeted phase-out scenarios using the syndicate substitute rule. The equivalent using the any substitute rule is shown in the main text as Figure 4. Results are qualitatively equivalent for both substitution rules with the exception of the size of the efficiency gap, which is larger in the targeted scenario than the random scenario. This counter-intuitive result arises since the largest banks are also those with the largest number of syndicate partners, and they are therefore more likely to successfully substitute capital than the smaller banks, delaying the onset of the tipping point relative to the any substitute scenario. This effect, combined with the survival of peripheral deals funded by smaller banks which survive for an extended duration in the targeted scenario, means that the phase-out of finance is less effective in this instance.

(ii) Limiting the number of candidate banks for substitution

In the syndicate substitute model, a deal at risk will attempt to acquire substitute finance from any of the banks which are co-active with the current syndicate. Here, we adapt the model to limit the number of possible banks who can act as a candidate for substitution. If a deal at risk fails to acquire substitute finance from those limited candidate banks, the deal fails. Results with two finance limits (10% and 50% for illustrative purposes) are shown in Fig. S7.

Figure S7a shows that imposing limits to the number of substituting partners reduces the number of banks which must phase-out of the sector to reach the tipping point. In particular, if the limit on the candidate banks is highly restrictive, this can substantially improve phase-out efficiency. However, the key feature of the model – a sharp transition from low to high phase-out efficiency – persists in this variant. The efficiency gap and tipping point both decrease with tighter limits on the number of substitute banks (Fig. S7 b & c). However, phase-out efficiency remains low, even with tight limits on the number of substitute banks, unless strict finance limits are also in place.

(iii) Limiting the number of times a deal can acquire substitute finance

In the base model, there is no limit to the number of times a single deal can substitute finance when a syndicate partner phases-out from the fossil fuel sector. In this model variant we incorporate a feature of reflecting the perceived 'risk' of a deal whereby a deal can only substitute finance a certain number of times before automatically failing. Results are shown in Fig. S8 for two fixed values of the finance limit and variable limits to the number of times a single deal can substitute finance. Simulations use the syndicate substitute model variant.

Fig. S8a shows that limiting the number of times a deal can acquire substitute finance significantly increases phase-out efficiency so that fewer banks are required to reach the tipping point. Note however, that Fig. S8b shows that limits to the number of times a deal can acquire substitute finance are only effective if the limit is very small (1 or 2 substitutions), and that this effect is secondary to restrictions on finance limits.

(iv) Defining the finance limit in terms of absolute exposure to the fossil fuel sector.

Figure S9 shows the phase-out model using the any substitute rule and with banks phased-out in a random order in which the limit to a bank's fossil fuel investments is set as a percentage of the banks total exposure to the sector (fossil fuel investments from a given year as a percentage of total assets). Exposure values are only available for the top 30 banks. Consequently, we model the exposure of other banks to the sector according to a gamma distribution which is fitted to the known data for the largest thirty banks. With a non-zero exposure limit, this model exhibits an efficiency gap, and a tipping point from inefficient to efficient phase-out which is a function of the stringency of the limit imposed.

(v) Allowing the reassignment of finance from failed deals to new deals via substitution

All models thus far assume that if a deal fails to acquire substitute finance, any finance committed to the deal by banks which remain active in the fossil fuel sector cannot be reassigned to new deals. In this way, the direct phase-out of finance can induce the indirect phase-out of finance. Figure S10 shows that the base model is robust to allowing the reassignment of finance to new deals, i.e., a bank which previously had $1bn assigned to fossil fuel deal A which has now failed (due to the failure to substitute finance) can use this finance to provide substitute finance to another deal-at-risk, deal B, if the bank is not already a syndicate partner in deal B. In this version of the model, we see the emergence of an efficiency gap and note that the tipping point from inefficient to efficient phase-out is a function of the stringency of the finance limit. Note, however, that because finance can be reassigned, direct phase-out does not induce indirect phase-out, and therefore we do not observe a phase-out multiplier larger than 1. This means that for the same finance limit percentage, the model

with finance reassignment has a larger efficiency gap and a later tipping point than the base model where finance cannot be reassigned.

References

1. Beyene, Winta, Manthos D. Delēs, and Steven Ongena. *Financial Institutions' Exposures to Fossil Fuel Assets: An Assessment of Financial Stability Concerns in the Short Term and in the Long Run, and Possible Solutions: Study Requested by the ECON Committee*. European Parliament, 2022.
2. Cojoianu, Theodor F., et al. 2023. *"The city never sleeps: but when will investment banks wake up to the climate crisis?."* Regional Studies, 57(2).
